# Tunneling Nanotubes-Mediated Protection of Mesenchymal Stem Cells: An Update from Preclinical Studies

**DOI:** 10.3390/ijms21103481

**Published:** 2020-05-14

**Authors:** Thangavelu Soundara Rajan, Agnese Gugliandolo, Placido Bramanti, Emanuela Mazzon

**Affiliations:** 1 Department of Biotechnology, School of Life Sciences, Karpagam Academy of Higher Education, Coimbatore 641021, India; tsrajanpillai@gmail.com; 2 IRCCS Centro Neurolesi “Bonino-Pulejo”, Via Palermo, Contrada Casazza S.S.113, 98124 Messina, Italy; agnese.gugliandolo@irccsme.it (A.G.); placido.bramanti@irccsme.it (P.B.)

**Keywords:** tunneling nanotubes, mesenchymal stem cells, mitochondria, cancer cells, preclinical studies

## Abstract

Tunneling nanotubes (TNTs) are thin membrane elongations among the cells that mediate the trafficking of subcellular organelles, biomolecules, and cues. Mesenchymal stem cells (MSCs) receive substantial attention in tissue engineering and regenerative medicine. Many MSCs-based clinical trials are ongoing for dreadful diseases including cancer and neurodegenerative diseases. Mitochondrial trafficking through TNTs is one of the mechanisms used by MSCs to repair tissue damage and to promote tissue regeneration. Preclinical studies linked with ischemia, oxidative stress, mitochondrial damage, inflammation, and respiratory illness have demonstrated the therapeutic efficacy of MSCs via TNTs-mediated transfer of mitochondria and other molecules into the injured cells. On the other hand, MSCs-based cancer studies showed that TNTs may modulate chemoresistance in tumor cells as a result of mitochondrial trafficking. In the present review, we discuss the role of TNTs from preclinical studies associated with MSCs treatment. We discuss the impact of TNTs formation between MSCs and cancer cells and emphasize to study the importance of TNTs-mediated MSCs protection in disease models.

## 1. Introduction

Tunneling nanotubes (TNTs) are thin and long membrane elongations among the cells. They consist of F-actin and are about 50–1000 nm in width and 100 µm in length [1]. TNTs represent a novel form of intercellular connection for a longer distance through which calcium, proteins, microRNAs, subcellular organelles, and cellular vesicles are transferred from one cell to another cell [2]. Mesenchymal stem cells (MSCs) are stromal cells, nonhematopoietic in nature, and can be differentiated into multiple cell lineages. They can be isolated from dental tissue, bone marrow, adipose tissue, umbilical cord, placenta, and other resources. A variety of MSCs-secreted immunomodulatory factors and growth factors has been documented via exosomes/microvesicles [3]. Extensive preclinical studies have established the therapeutic role of MSCs and/or their secretome in different disease models. The secretory factors and the absence of major histocompatibility complex II (MHCII) and factors associated with T cells coactivation enables MSCs theoretically suitable for allogeneic transplantation in clinical studies [4]. MSCs exhibit their therapeutic efficacy in tissue engineering and regenerative medicine through four potential mechanisms: i) direct cell-to-cell signaling; ii) paracrine signaling with soluble secreted factors such as hormones and proteins; iii) homing of released exosomes or microvesicles that contain immunoregulatory molecules and other molecules including RNA; iv) mitochondrial trafficking via TNTs or microvesicles [5]. Apart from preclinical studies associated with ischemia, oxidative stress, mitochondrial damage, inflammation, and respiratory illness, MSCs formed TNTs with cancer cells where it has been demonstrated that TNTs enhance the chemoresistance property of tumor cells.

Despite a novel mechanism, a limited number of in vitro and in vivo studies have reported the TNTs-based mechanism of action of MSCs.

In the present review, we discuss TNTs-driven mitochondrial trafficking from preclinical models treated with MSCs. To collect research articles related to TNTs we performed PubMed and Google scholar searches using the following keywords: “Tunneling nanotubes” and “Mesenchymal stem cells”. We emphasize the importance to study the TNTs-based mechanism of action of MSCs in other preclinical models.

## 2. Preclinical Studies

### 2.1. Ischemia/Reperfusion Model

An *in vitro* study by Liu et al. observed that human bone marrow MSCs rescued human umbilical cord vein endothelial cells subjected to oxygen and nutrients deprivation-induced stress, an *in vitro* ischemia/reperfusion model. Apoptosis in the endothelial cells induced by dysfunctional mitochondria was abolished by shifting of the functional mitochondria from MSCs through TNTs-like cell protrusions [6]. A similar anti-apoptotic effect of TNTs-mediated mitochondrial transfer was noticed in oxidative stress-induced H9c2 cardiomyocytes treated with rat bone marrow MSCs [7].

An interesting study by Figeac et al. demonstrated that the oxidative stress microenvironment of the mouse cardiomyocytes triggered human adipose MSCs to secrete factors related to cardiac protection. Soluble factors including hepatocyte growth factor (HGF), vascular endothelial growth factor (VEGF), stromal cell-derived factor 1-alpha (SDF-1α), and monocyte chemotactic protein-3 (MCP-3) were increasingly released by MSCs into the mouse cardiomyocytes by TNTs. Moreover, engraftment of human adipose MSCs preconditioned with distressed mouse cardiomyocytes significantly increased the cardiac function with elevated angiogenesis in mice subjected to myocardial infarction, suggesting TNTs-driven protective mechanism of MSCs [8].

An *in vitro* study by Babenko et al. reported that human bone marrow MSCs rescued rat astrocytes and neuron-like PC12 pheochromocytoma cells from oxygen–glucose deprivation-induced oxidative stress and mitochondrial damage, respectively. *In vivo* data from the same study showed that MSCs ameliorated the neurological impairments of cerebral ischemic rats. Data from this study demonstrated that the mitochondrial impairment was nullified by the transfer of mitochondria from bone marrow MSCs via TNTs, which in turn re-established the bioenergetics of the damaged cells. It was found that the degree of mitochondrial transfer from MSCs was more efficient when injured astrocytes were associated with an elevated reactive oxygen species level. In the same study, it was reported that ischemia rats treated with bone marrow MSCs that overexpressed the mitochondrial Rho-GTPase 1 protein (Miro1) showed significant improvement. Miro1 is a calcium-dependent adaptor protein that favors mitochondrial trafficking through microtubules and TNTs [9].

A recent *in vivo* study showed enhanced activity of mitochondria which resulted in angiogenesis progression, microvasculature restoration, and improved neurological activity in ischemic stroke injured rats administered with rat bone marrow MSCs. TNTs-mediated mitochondrial transfer was noticed in the cerebrovascular system of ischemic rats exposed to MSCs engrafting [10]. Another recent *in vitro* study reported two formation stages of TNTs between human MSCs derived from the umbilical cord and oxidative stress-induced neonatal mouse cardiomyocytes. In the active formation stage (≤16 h in coculture), more TNTs were protruded from MSCs while in the mature and stable stage (>16 h in coculture), more TNTs were protruded from distressed cardiomyocytes. It was observed that TNTs-mediated mitochondrial transfer from MSCs inhibited the hypoxia-based apoptosis in cardiomyocytes only in the mature and stable stage, assuming morphological characteristics of TNTs may play a crucial role in mitochondrial transfer [11]. These data supported the earlier *in vitro* MSCs-rat neonatal cardiomyocytes coculture study reported by Yang et al. In this study, it was observed that the initial TNTs formation was derived from MSCs while after 24 h of coculture, majority of TNTs (67%) were originated from rat cardiomyocytes. However, mitochondrial trafficking was unidirectional from MSCs TNTs. Interestingly, the authors found a small number of TNTs between rat cardiomyocytes and cardiac fibroblasts with no mitochondrial shift, suggesting the differential ability of cells to generate TNTs with other interconnected cells [12].

### 2.2. Chemotherapy and Other Stress-Induced Models

Feng et al. reported that human bone marrow MSCs recovered the hematopoietic potency of human umbilical cord endothelial cells induced with cytarabine in an *in vitro* chemotherapy-based stress model. The observed apoptosis reduction in endothelial cells was linked with the mitochondrial transfer of MSCs via TNTs. Simultaneously, the TNTs-mediated shift of mitochondria restored transmembrane migration and capillary angiogenic potency of endothelial cells exposed to cytarabine [13]. A study by Jiang et al. revealed that direct coculture of human-induced pluripotent stem cells (iPSC)-derived MSCs nullify the oxidative stress induced by rotenone in corneal epithelial cells through TNTs-based mitochondrial transfer. The results showed that the TNTs formation was related to nuclear factor κB-TNFα induced protein 2 signaling pathway triggered by oxidative inflammation in corneal epithelial cells. In addition, TNTs-mediated mitochondrial transfer was observed *in vivo* study where MSCs cultured on a corneal scaffold was transplanted to a rabbit corneal injury model developed by sodium hydroxide administration [14].

In another *in vitro* study, human adipose derived MSCs exposed to antioxidants N-acetyl-L-cysteine (NAC) and L-ascorbic acid 2-phosphate (AAP) rescued MSCs from reduced mitochondrial density and membrane potential, caused by hydrogen peroxide-induced oxidative stress via TNTs-based mitochondrial transfer. The expression of mitochondrial fragmentation protein, dynamin-related protein 1 (DRP1S616), was blocked by the transferred mitochondria [15].

### 2.3. Stress Model from MELAS Patients

A recent study reported that MSCs provide healthy mitochondria to the cells derived from mitochondrial myopathy, encephalomyopathy, lactic acidosis, and stroke-like episodes (MELAS) patients. MELAS is an infrequent genetic disease associated with mitochondrial dysfunction. This mitochondrial disease is developed by the occurrence of point mutation in the mitochondrial *tRNALeu(UUR)* gene. In that study, it was observed that human Wharton’s jelly mesenchymal stem cells (WJMSCs) protected human MELAS fibroblasts from mitochondrial stress elicited by rotenone through mitochondrial transfer by TNTs. Impairments in the mitochondrial respiratory chain including low bioenergetics and membrane potential induced by rotenone in MELAS fibroblasts were rectified by the healthy mitochondria of WJMSCs. Moreover, the results showed that inhibiting the actin polymerization by cytochalasin B resulted in blocking TNTs formation which terminated the mitochondrial shift from MSCs. These data highlighted the pivotal role of TNTs formation in MSCs-based protection [16].

### 2.4. Inflammatory and Respiratory Disease Models

Transfer of rat bone marrow MSCs mitochondria through TNTs was noticed in rat nucleus pulposus cells subjected to inflammation by interleukin 1β exposure. Inflammation-induced apoptosis was suppressed by TNTs-mediated mitochondria movement from MSCs. It is imperative to mention that MSCs-based cell protection was observed only in the direct coculture method and not in the indirect coculture method, suggesting the key role of TNTs between MSCs and inflamed nucleus pulposus cells [17]. These data supported an earlier study where human bone marrow MSCs were found to connect with human nucleus pulposus cells via TNTs on a direct coculture system which resulted in phenotypic changes in the latter. However, in this study, neither MSCs nor pulposus cells were subjected to any inflammatory induction or other modes of stress [18]. In another *in vitro* study, unidirectional mode of cell-to-cell connection was observed between human MSCs and human vascular smooth muscle cells (VSMCs) in which mitochondria were transferred from VSMCs to MSCs through TNTs, and resulted in the increased proliferation of MSCs. No induction of MSCs differentiation was observed [19]. On the other hand, bi-directional cytoplasmic contents exchange was reported between human adipose derived MSCs and human peripheral T lymphocytes where TNTs were selectively originated from T cells [20]. These data suggested that TNTs formation and the cell-to-cell connection may be unidirectional or bi-directional and may depend on the microenvironment as well as the physiological nature of the interconnected cells.

In an *in vivo study*, administration of bone marrow MSCs from mouse and human displayed protective effect against mouse acute lung injury model via mitochondrial transfer by TNTs and microvesicles. In this study, it was demonstrated that the formation of TNTs and microvesicles was attributed to the Connexin-43 dependent gap junctional channels formed between MSCs and mouse alveolar epithelial cells [21]. Sinclair et al. investigated the TNTs formation capacity of MSCs derived from digested parenchymal lung tissue from healthy donors and bronchoalveolar lavage fluid derived from lung transplant recipient donors with human bronchial epithelial BEAS-2B cells. The results showed the formation of TNTs between MSCs and epithelial cells and that inhibiting the TNTs formation resulted in the dysregulated transfer of mitochondria and cytosolic contents into epithelial cells [22]. Ahmad et al. reported the protective effect of human bone marrow MSCs in *in vivo* asthma models of rotenone-induced airway epithelial injury and allergic airway inflammation and *in vitro* mice bronchial epithelial cells treated with conditioned medium derived from macrophages exposed to IL-13. The TNTs-mediated mitochondrial transfer was attributed to the protection elicited by MSCs. Increased mitochondrial transfer and anti-inflammatory responses were elicited by MSCs that overexpressed Miro1 protein. In addition, it was observed that shRNA mediated knockdown of tumor necrosis factor alpha induced protein 2 (TNFAIP2), a protein associated with TNTs formation, showed reduced TNTs synthesis by MSCs followed by decreased mitochondrial transfer into the epithelial cells [23].

Li et al. reported the protective efficacy of human iPSC and bone marrow MSCs in *in vitro* and *in vivo* model of cigarette smoke-induced lung damage. They noticed the transfer of mitochondria through TNTs by which MSCs attenuate the mitochondrial dysfunction in BEAS-2B cells and suppress the degree of alveolar destruction in the lungs of rats injured by cigarette smoke [24]. A study by Jackson et al. described the therapeutic effect of human bone marrow MSCs in *Escherichia coli*-induced *in vitro* and *in vivo* model of acute respiratory distress syndrome by TNTs-mediated mitochondrial transfer. Decreased level of inflammation and increased level of alveolar macrophages-driven phagocytosis, bacterial clearance, and functional recovery of injured lung tissue were noticed due to mitochondrial transfer from MSCs TNTs. Several pro-inflammatory cytokines including interleukin-1β, interleukin 6, tumor necrosis factor alpha (TNF-α), and Eotaxin were reduced while anti-inflammatory cytokines such as interleukin 4 and RANTES were increased after the mitochondrial transfer from MSCs [25]. It is imperative to mention here that in addition to TNTs, mitochondria and their components transfer may occur via exosomes/microvesicles secreted from MSCs into macrophages and other cells. Exosomes-mediated transfer of mitochondria has been reviewed elsewhere [26,27].

Yao et al. recorded the therapeutic effect of human iPSC-derived MSCs in mice subjected to asthmatic inflammation by ovalbumin treatment. TNTs-mediated mitochondrial transfer of MSCs significantly reduced the mitochondrial impairment as well as the expression of pro-inflammatory cytokines profile including interleukins 5, 13, and 33. In the same study, TNTs-mediated mitochondrial transfer of MSCs rescued the mitochondrial dysfunction in human BEAS-2B cells treated with cobalt (II) chloride (CoCl_2_). In addition, data from this study showed that the expression of gap junction protein connexin 43 plays a crucial role in the formation of TNTs from MSCs [28].

### 2.5. Other Non-Cancer Models

TNTs-based bi-directional exchange of cytoplasmic components was observed between human MSCs and rat renal tubular cells. However, substantial transport occurred in the MSCs from rat cells. The presence of Tamm–Horsfall protein, a glycoprotein uniquely synthesized by renal tubular cells, in MSCs suggested the possibility of differentiation of MSCs into renal tubular cells [29]. CoCl_2_-induced mitochondrial dysfunction in PC12 cells was recovered by human iPSC-derived MSCs. Mitochondrial transfer from MSCs by TNTs significantly nullified the distressed mitochondrial indexes such as swelling and cristae degeneration in PC12 cells [30].

### 2.6. Cancer Models

TNTs-mediated mitochondrial transfer from MSCs protected cancer cells from death induced by drugs which is an undesired event. Exchange of cytoplasmic components through TNTs was observed between human stromal cells including MSCs and human cancer cell lines such as SKOV3 ovarian cancer cell line and MCF7 breast cancer cell line [31]. An important study by Polak et al. demonstrated TNTs establishment between primary B-cell precursor acute lymphoblastic leukemia (BCP-ALL) cells and human bone marrow MSCs. Prosurvival cytokines including IL 8 and MCP1/CCL2 were secreted by MSCs into BCP-ALL cells via TNTs signaling, which resulted in chemoresistance of BCP-ALL cells against prednisolone, a glucocorticoid drug used in cancer therapy to induce apoptosis [32].

Marlein et al. showed that the presence of NADPH oxidase 2-induced superoxide in human acute myeloid leukemia (AML) cells primed the human bone marrow MSCs to shift the mitochondria from the latter via TNTs in an in vitro coculture setup [33]. Recently, the same group demonstrated CD-38 dependent TNTs formation between human multiple myeloma cells and human bone marrow MSCs [34].

Jurkat cell line, a model for human primary T cell-ALL (T-ALL), reportedly found to exchange mitochondria by TNTs upon exposure to cancer chemotherapeutic drugs (methotrexate and cytarabine) with human bone marrow MSCs. As a result, Jurkat cells attained chemoresistance from drug-induced apoptosis. The TNTs-mediated mitochondrial transfer was abolished when the cells were exposed to an antibody, which neutralized the activity of intercellular adhesion molecule 1 [35]. A recent study reported that human bone marrow MSCs activated by reactive oxygen species rescued human acute lymphoblastic leukemia (ALL) cell lines from drug-induced apoptosis and cell death. The rescue was attributed to TNTs-based mitochondrial transfer from activated MSCs [36].

**Figure 1:**
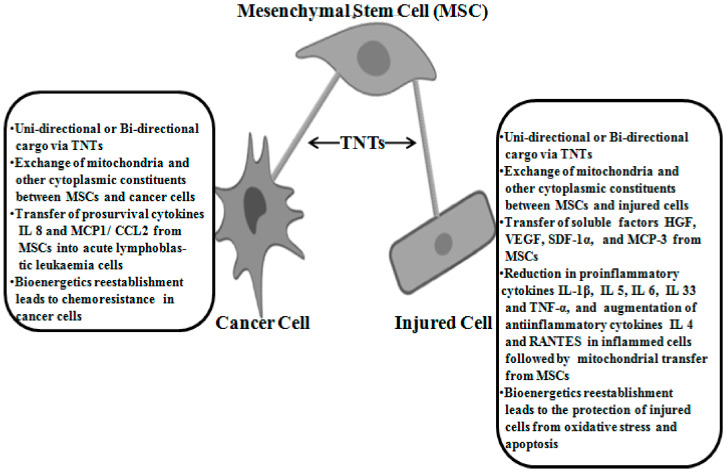
Tunneling nanotubes (TNTs)-mediated effects of mesenchymal stem cells in injured cells and cancer cells.

A summary of the study reported in this review is available in Table 1. The major effects of TNTs-mediated action of MSCs with injured and cancer cells are highlighted in Figure 1.

## 3. Future Directions

TNTs-mediated protective mechanism of MSCs has been demonstrated predominantly in preclinical models associated with ischemia/reperfusion, mitochondrial dysfunction, oxidative stress, and asthmatic inflammation. MSCs showed enormous therapeutic efficiency against a wide variety of diseases. MSCs-based clinical trials have been increasing day by day [37]. Of note, TNTs-based intercellular communication has been described in the central nervous system [38]. The putative role of the TNTs-driven mitochondrial shift has been studied in rat cerebral ischemia model administered with human bone marrow MSCs [39]. Nevertheless, the primordial role of TNTs-mediated intercellular connection by MSCs has not been elucidated in many diseases including neurodegenerative diseases and autoimmune diseases. It is important to understand the possible exchange of mitochondria and other subcellular organelles and other secreted factors through TNTs in these disease microenvironments treated with MSCs. Accordingly, future studies shall be projected towards defining the role of TNTs in MSCs-treated disease models including neurodegenerative diseases and autoimmune diseases. Given the unidirection and bi-direction mode of the TNTs-based cargo among cells, a study on the nature of microenvironment that favors TNTs establishment in different types of cells might help to extend the results to the clinical level. MSCs in cancer therapy remains elusive as there are reports for both therapeutic potency and cancer progression [40]. As TNTs are protruded from MSCs as well as cancer cells it is indispensable to understand the cues generated from both cells using TNTs as a bridge which eventually determines what subcellular contents or secreted factors shall be transferred and in what direction the cargo is. We believe that results from these future preclinical studies based on above-mentioned themes may have key impacts on MSCs-based clinical trials.

## 4. Conclusions

TNTs are a novel cargo route among cells through which subcellular organelles and secreted factors are transferred. Preclinical studies have demonstrated the TNTs-mediated protection of MSCs in a wide range of stress microenvironments. Mitochondria transfer from MSCs is predominant through TNTs which reframe the cancer cells to attain chemoresistance against drugs. Further preclinical studies are warranted to understand the key mechanisms underlying TNTs-driven protection of MSCs against a wide array of diseases that might be translated into clinical studies.

## Figures and Tables

**Table 1:** Overview of the studies reported in this review regarding tunneling nanotubes (TNTs) trafficking in preclinical models associated with mesenchymal stem cells (MSCs) treatment.

Field of Study	MSCs Source	Experimental Model	Results	Ref.
Ischemia/reperfusion	Human bone marrow MSCs	Human umbilical vein endothelial cell exposed to OGD	Reduced apoptosis	[6]
Ischemia/reperfusion	Rat bone marrow MSCs	H9c2 rat ventricular cell line *in vitro* model of SI/R	Reduced apoptosis	[7]
Ischemia/reperfusion	Human adipose MSCs	*In vitro*: Mouse cardiomyocyte; *In vivo*: mouse subjected to MI	*In vitro*: increased release of cardioprotective soluble factors by MSCs; *In vivo*: greater cardiac function recovery associated with higher angiogenesis	[8]
Ischemia/reperfusion	Human bone marrow MSCs	*In vitro*: Rat astrocytes exposed to OGD and PC12 pheochromocytoma cells exposed to ethidium bromide; *In vivo*: Rats subjected to MCAO	*In vitro*: MSCs rescued astrocytes and PC12 cells from damage; *In vivo*: MSCs, especially those overexpressing Miro1, ameliorated neurological impairment	[9]
Ischemia/reperfusion	Rat bone marrow MSCs	Rats subjected to MCAO	Increase in angiogenesis, improvements in functional recovery	[10]
Ischemia/reperfusion	Human umbilical cord MSCs	Neonatal mouse cardiomyocytes exposed to hypoxia/reoxygenation	Reduced apoptosis	[11]
Ischemia/reperfusion	Rat bone marrow MSCs	Neonatal rat cardiomyocytes	Initial TNTs formation derived from MSCs, while after 24h majority of TNTs derived from cardiomyocytes	[12]
Chemotherapy	Human bone marrow MSCs	Human umbilical cord vein endothelial cells treated with cytarabine	Reduced apoptosis, restored transmembrane migration ability and angiogenic capacity of endothelial cells	[13]
Oxidative stress	Human iPSC-derived MSCs	*In vitro*: Corneal epithelial cells exposed to rotenone; *In vivo*: Rabbit model of corneal alkaline burn induced by sodium hydroxide	*In vitro*: protection against rotenone oxidative stress; *In vivo*: beneficial effects for corneal wound recovery	[14]
Oxidative stress	Human adipose derived MSCs	MSCs exposed to hydrogen peroxide, N-acetyl-L-cysteine, and L-ascorbic acid 2-phosphate	Antioxidants increased mitochondrial mass and respiratory capacity	[15]
MELAS patients	Wharton’s jelly MSCs	Human MELAS fibroblasts treated with rotenone	Improved mitochondrial stress	[16]
Inflammation	Rat bone marrow MSCs	Rat nucleus pulposus cells exposed to IL-1β	Reduced apoptosis only in direct coculture	[17]
	Human bone marrow MSCs	Human nucleus pulposus cells	TNTs formation in coculture system resulting in phenotypic changes in human nucleus pulposus cells	[18]
Inflammatory disease	Human bone marrow MSCs	Human vascular smooth muscle cells	Increased MSCs proliferation, but not differentiation	[19]
Immunomodulation	Human adipose derived MSCs	Human peripheral T lymphocytes	Bi-directional cytoplasmic content exchanges	[20]
Lung injury	Mouse and human bone marrow MSCs	Mouse acute lung injury model	Mitochondria transfer by TNTs exerted protective effects	[21]
Lung injury	Human MSCs derived from digested parenchymal lung tissue and from lung transplant recipients’ bronchoalveolar lavage fluid	Human bronchial epithelial BEAS-2B cells	Formation of TNTs between MSCs and epithelial cells	[22]
Lung injury	Human bone marrow MSCs	*In vivo*: Mouse model of rotenone-induced airway epithelial injury and allergic airway inflammation *In vitro*: Mouse bronchial epithelial cells treated with conditioned medium derived from macrophages exposed to IL-13	Protection exerted by TNTs mitochondrial transfer. MSCs overexpressing Miro1 showed increased mitochondria transfer	[23]
Respiratory disease	Human iPSC-derived MSCs and bone marrow MSCs	*In vitro*: Human bronchial epithelial BEAS-2B cells exposed to cigarette smoke medium *In vivo*: Rats injured by cigarette smoke	*In vitro*: attenuated mitochondrial dysfunction *In vivo*: improved alveolar destruction	[24]
Respiratory disease	Human bone marrow MSCs	*In vitro*: Human monocyte-derived macrophages exposed to LPS or *Escherichia coli* *In vivo*: Mice subjected to *Escherichia coli* pneumonia	*In vitro*: enhanced phagocytic capacity; *In vivo*: enhanced alveolar macrophages phagocytosis, decreased inflammation, and functional recovery	[25]
Respiratory disease	Human iPSC-derived MSCs	*In vivo*: Mice subjected to asthmatic inflammation by treatment with ovalbumin; *In vitro*: Human bronchial epithelium cell line BEAS-2B cells treated with CoCl_2_	*In vivo*: Attenuated mitochondrial dysfunction and inflammation; *In vitro*: Attenuated mitochondrial dysfunction	[28]
	Human MSCs	Rat renal tubular cells	Bi-directional cytoplasmic content exchanges and MSCs differentiation	[29]
Mitochondria damage	Human iPSC-derived MSCs	PC12 cells treated with CoCl_2_	Attenuated mitochondrial dysfunction	[30]
Cancer	MSCs	Human cancer cell lines, such as SKOV3 ovarian cancer cells and MCF7 breast cancer cells	Exchange of cytoplasmic components	[31]
Cancer	Human bone marrow MSCs	Primary B cell precursor acute lymphoblastic leukemia cells (BCP-ALL)	Chemoresistance of BCP-ALL to prednisolone	[32]
Cancer	Human bone marrow MSCs	Acute myeloid leukemia cells	NADPH oxidase-2 derived superoxide drove mitochondrial transfer from MSCs to leukemic cells	[33]
Cancer	Human bone marrow MSCs	Human multiple myeloma cells	CD-38 dependent TNTs formation	[34]
Cancer	Human bone marrow MSCs	Human T cell acute lymphoblastic leukemia cell line Jurkat exposed to methotrexate and cytarabine	Chemoresistance from drug-induced apoptosis	[35]
Cancer	Human bone marrow MSCs	Human acute lymphoblastic leukemia cell lines	Reduced drug-induced apoptosis	[36]

CoCl_2_, Cobalt (II) chloride; iPSC, induced pluripotent stem cells; MCAO, middle cerebral artery occlusion; MELAS, Mitochondrial myopathy, Encephalopathy, Lactic acidosis, and Stroke-like episodes; MI, myocardial infarction; Miro1, Mitochondrial Rho-GTPase 1; MSCs, mesenchymal stem cells; OGD, oxygen–glucose deprivation; SI/R, simulated ischemia/reperfusion.

## Abbreviations

TNTs	Tunneling nanotubes
MSCs	Mesenchymal stem cells
MHCII	Major histocompatibility complex II
HGF	Hepatocyte growth factor
VEGF	Vascular endothelial growth factor
SDF-1α	Stromal cell-derived factor-1α
MCP-3	Monocyte chemotactic protein-3
Miro1	Mitochondrial Rho-GTPase 1
iPSC	Induced pluripotent stem cells
NAC	N-acetyl-L-cysteine
AAP	L-ascorbic acid 2-phosphate
DRP1	Dynamin-related protein 1
MELAS	Mitochondrial myopathy, Encephalopathy, Lactic acidosis, and Stroke-like episodes
WJMSCs	Wharton’s jelly mesenchymal stem cells
VSMCs	Vascular smooth muscle cells
TNF-α	Tumor necrosis factor-alpha
CoCl_2_	Cobalt (II) chloride
BCP-ALL	B-cell precursor acute lymphoblastic leukemia
AML	Acute myeloid leukemia
T-ALL	T cell acute lymphoblastic leukemia
